# Forging partnerships for health equity research: transformative capacity-building for community-academic teams

**DOI:** 10.3389/fpubh.2025.1617711

**Published:** 2025-07-29

**Authors:** Carmen R. Valdez, Liana Petruzzi, Phillip W. Schnarrs, Tasha Banks, Chris M. Coombe, Barbara A. Israel

**Affiliations:** ^1^Department of Health, Behavior and Society, UT School of Public Health San Antonio, San Antonio, TX, United States; ^2^Department of Health Studies, College of Liberal Arts, American University, Washington, DC, United States; ^3^School of Education, American University, Washington, DC, United States; ^4^Department Behavioral and Community Health Science, School of Public Health, University of Pittsburgh, Pittsburgh, PA, United States; ^5^Center for LGBT Health Research, School of Public Health, University of Pittsburgh, Pittsburgh, PA, United States; ^6^St. David’s Foundation, Austin, TX, United States; ^7^Detroit Community-Academic Urban Research Center, Ann Arbor, MI, United States; ^8^Department of Health Behavior and Health Equity, School of Public Health, University of Michigan, Ann Arbor, MI, United States

**Keywords:** health equity, continuing education, community-based participatory research (CBPR), capacity-building, partnerships & collaborations

## Abstract

**Background:**

Community-based participatory research (CBPR) is essential for translating and increasing the overall uptake of evidence-based interventions in community settings. Yet a limited number of academic and medical institutions provide structured and formal training on how to conduct high-quality CBPR or develop academic-community partnerships.

**Methods:**

Building upon a capacity-building program, we developed and implemented a year-long academic partnership training program. It consisted of a 2.5-day intensive short course, bimonthly didactic webinars, and year-long mentoring, as well as seed funding. Five dyads of academic researchers from universities in Texas and their community partners completed the program (*n* = 10) between May 2023 and May 2024. A mixed methods evaluation via a survey with closed- and open-ended questions was conducted at the end of the 12 months to evaluate satisfaction with the program and impact.

**Results:**

Nine out of the ten participants reported the program was excellent or very good, and all participants found the mentoring component and didactic sessions to be “transformative.” Participants highlighted the importance of forming close relationships with their partners and other teams, peer mentorship, and having a space to discuss challenges associated with CBPR. Both academic and community participants reported making significant progress on their research projects including local, state and federal conference presentations, applying for and securing grant funding, and submitting peer-reviewed manuscripts. They created tools that were helpful for their community.

**Discussion:**

Dedicated training in CBPR practices for community practitioners and academics in the same space can build capacity for health equity research and initiatives. Using a combination of didactic and experiential learning opportunities, in addition to peer and formal mentorship, allowed for considerable growth among participants. Importantly, academics developed understanding and interest in community projects, and community members developed interest in research and appreciation for academic institutions. Suggestions for improving the program are also discussed.

## Introduction

Community-based participatory research (CBPR) is essential for implementing effective health equity initiatives (1). CBPR is an approach to research that intentionally shares power with community partners and involves them in the research process with the goal of greater benefit to the community (2). By fostering collaboration between local community leaders and researchers, CBPR equips community leaders with rigorous academic tools and helps secure funding for scaling up initiatives. At the same time, it enables researchers to align their priorities with those of the community, leverage local knowledge, and engage stakeholders across systems, including residents, practitioners, and policymakers (1). This reciprocal approach compels research to be actionable and enhances its sustainability (2). Despite these compelling reasons for CBPR, the current academic and research enterprises are not adequately incentivized to build and maintain engaged research capacity among researchers, communities or health systems (3). NASEM puts forth several recommendations for improving community engagement research including workforce capacity and training, institutional structures and faculty incentives (1).

Underscoring the need for equitable community engagement is the stark fact that traditional research paradigms (e.g., RCTs in highly controlled settings) in the absence of meaningful community buy-in have failed to significantly reduce health disparities among vulnerable populations (4, 5). Moreover, reviews of existing discoveries demonstrated that it takes approximately 17 years for 14% of original research to translate into public health practice (6). CBPR is one solution for improving the translation of evidence-based practices, as it includes implementation of internal and external processes that are essential for research translation, such as community readiness and fit between innovation and community preferences.

Academic institutions are called to develop the infrastructure for CBPR, yet incorporating community members with lived experience requires meaningful engagement (7). Moreover, there is considerable community distrust for academic and medical institutions due to historical and ongoing harms (8, 9). Clinical and Translational Science Award centers often address these limitations through structured interactions between faculty seeking research input, and community members with lived experience, for example Community Engagement Studios (10, 11). Those programs, though, are meant to include community members as advisors, not as collaborators, in the research. Deeper engagement is needed to position community members as equal partners in the research (7).

There are few notable examples of capacity-building for academic-community collaborators. One program, *Engage for Equity*, created tools to support community–academic partnerships in strengthening their research processes and outcomes (12). The tools, informed by prior studies conducted by Wallerstein and colleagues (13) aim at guiding (a) reflection about the partnership context and history, (b) planning and evaluation of partnership processes and intervention implementation, (c) strengthening partnership quality, and (d) promising practices. One such tool is the River of Life Reflection, where teams represent their collective partnership journey through artistic expression, identifying accomplishments, turning points, and roadblocks. The activity relies on symbols, drawings, magazine clippings, and any other type of visual representation to use as metaphors for their partnership history and progress. Evaluations of this and the other tools show these tools are feasible and acceptable to participants (12). These tools have been used with hundreds of trainees as part of a summer institute, although it is offered for a fee to participants. While the institute is open to researchers, students, and community members, it is not clear whether the program is intended for teams that have both academics and community members who are committed to working together.

Another exemplary program is the *CBPR Partnership Academy* (CBPR Academy) conducted by the Detroit Community-Academic Urban Research Center (Detroit URC) since 1995. The CBPR partnership involves eight community-based organizations, two health and human service agencies, and three units at the University of Michigan, aimed at fostering and supporting CBPR partnerships to achieve health equity (14). The year-long CBPR Academy engages cohorts of approximately 12 community-academic teams in a free-of-cost 5-day course focused on (a) describing and understanding partnership formation and maintenance, (b) the use of qualitative, quantitative and mixed methods in the context of CBPR studies, (c) the use of mixed methods to evaluate partnership process and outcomes, and (d) dissemination and translation of partnership results (14). Beyond the course, ongoing learning activities include a small planning grant, six online forums with all teams, and individual meetings with a community-academic mentor team over the course of the year. A mixed-method evaluation with two cohorts highlighted that providing modeling and mentoring to teams of community members and their academic partners solidified their partnerships and strengthened their capacity to grow and sustain their initiatives (14).

In collaboration with the Detroit URC’s CBPR Academy facilitators, we developed and piloted a year-long training and professional development program entitled Community-Academic Partnerships for Health Equity Program (CAP-HEP) at our medical institution in Texas. The program was adapted in content and format for the specific needs of partnerships in our region. In this article, we describe the recruitment of community-academic teams, the team selection process, and the program components. We also report the outcomes of surveys with closed and open-ended questions intended to assess the program’s feasibility, acceptability, and impact.

## Materials and methods

### Team and program development

#### The CAP-HEP team

CAP-HEP was developed by the first four authors, who are faculty and administrative and research staff with a history of strong community partnerships throughout the region. The team developed CAP-HEP based on previous CBPR training as well as institutional efforts to support CBPR. For example, in 2020, the first author developed the CBPR Collaborative, which she co-led with the third author. The CBPR Collaborative consisted of monthly meetings with academic researchers from their institution who were engaged in community-based research and community-academic partnerships, and who sought input and collaboration on their CBPR grants and manuscripts. It was also open to faculty and staff with limited experience with CBPR but who were interested in starting a CBPR project. Additionally, in 2020 the first and second authors created a 6-session CBPR webinar series that they offered to faculty and postdoctoral fellows from the medical school and university at large to promote awareness of and build knowledge and skills in CBPR.

While the CBPR Collaborative and CBPR webinar series we created were focused on faculty capacity building for CBPR, they did not provide structured opportunities to learn with community partners. Recognizing that learning CBPR is best when it is applied (15), the team envisioned the CAP-HEP program would be for researchers who had established a new partnership, but lacked formal CBPR training, and their community partners. By involving teams of community-academic partners in the early stages of partnership development and engaging them over an extended period, CAP-HEP aimed to strengthen the trust essential to effective academic-community collaborations and to build the capacity of both partners to engage meaningfully in this work. The Detroit URC’s CBPR Academy was a model for CAP-HEP, with the first author having been a fellow of that program in years prior. The third author participated in a training that included the River of Life tool from *Engage for Equity*.

#### The planning process

The first author met with university leaders in the spring and summer of 2022 to seek funds, securing a commitment of $25,000 from a university grand challenge initiative focused on community health, and $100,000 from a local foundation. This funding was used to support faculty and staff salaries (59%), consultants (21%), and operational costs (e.g., hosting, supplies, seed funding for teams; 20%), which allowed us to feasibly launch the program with a first cohort of five academic-community partner teams from two universities. The local focus of the CAP-HEP program allowed for in person participation in more than half of the activities.

Planning for CAP-HEP began in the fall of 2022 with bi-monthly meetings focused on the scope of the program and budget, timeline of implementation, marketing and promotion, recruitment and selection of teams, and curriculum development. The team also used planning meetings to identify potential academic and community partners who could serve as training facilitators and co-mentors in the program. We sought input from a Community Strategy Team at our academic unit, composed of six community advocates and leaders whose role is to inform the school on community priorities and initiatives. These leaders gave input on the format of CAP-HEP and assisted with community outreach. Additionally, consultants from the Detroit URC’s CBPR Academy met with the team on a quarterly basis over a two-year period, where insights and materials from their own program were shared. As noted above, the Detroit URC, established in 1995, has a longstanding commitment to CBPR and fostering and supporting the development and sustainability of CBPR partnerships. The Detroit URC has been guided by a decision-making community-academic Board that for the past 30 years has met monthly to shape the Center’s priorities and initiatives. The CBPR Partnership Academy was one of the results of this priority- setting process by the Board and became a focus of their efforts starting in 2014. Both the community and academic partners on the Board played critical, equitable roles in designing the Academy, grant proposal writing, implementing the funded initiative, including serving as instructors, mentors, reviewers of grant proposals, and evaluators (14). Thus, the design and implementation of CAP-HEP in Central Texas was co-created and aligned with the Detroit URC’s CBPR principles from the outset.

**Figure 1:**
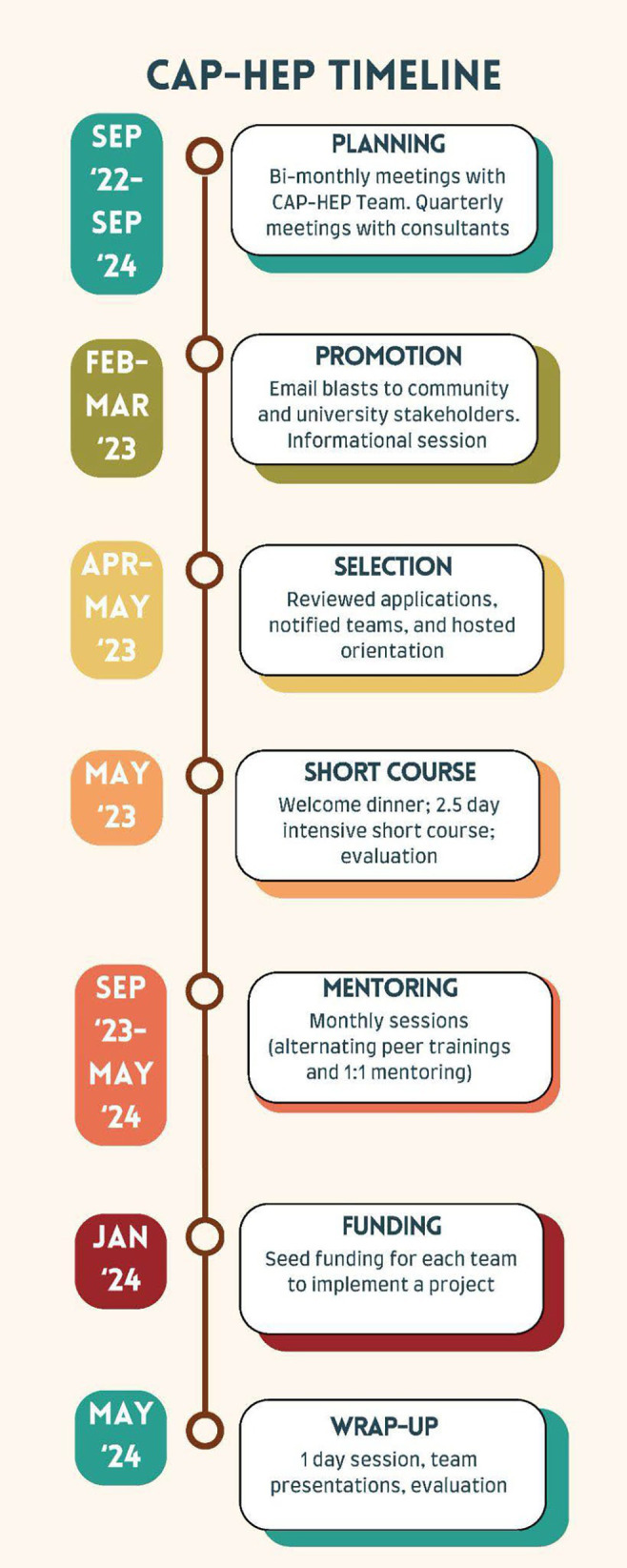
Timeline of CAP-HEP activities.

From these planning meetings, the CAP-HEP team decided on a 12-month CAP-HEP program that would largely mirror the CBPR Academy and include participation in a short-course, monthly peer mentoring and didactic meetings, one-on-one mentoring, and seed funds for project implementation and/or evaluation (see Figure 1 for a timeline). Packaging the program in this way aimed to foster and pace community-academic trust building, skill acquisition, and successful implementation of collaborative projects.

#### Recruitment and selection of community-academic partners

In the spring of 2023, the CAP-HEP team promoted the program through our division’s community-facing monthly newsletter and a flyer distributed directly to the Community Strategy Team and existing community collaborators. To recruit academic researchers, the CAP-HEP team shared the promotional materials with departments, schools and centers across campus. A virtual informational session was hosted in March for academics and community members interested in learning more about the CAP-HEP program. In April, the CAP-HEP team reviewed applications submitted by nine community-academic teams, selecting five partnership teams who met the following criteria: (a) team was in the early stages of partnership, (b) team had a proposed health equity project that was conceptualized by both the academic and the community partner, (c) team articulated how the CAP-HEP program would support their partnership, and (d) team submitted a commitment letter from their supervisors to optimize their ability to fully participate in the program. The five teams that were selected among the nine applicants, and their background and projects are described in Table 1.

**Table 1:** Community-academic team members and their projects.

Team	Academic partner	Community partner	Project focus
1	Assistant professor in nursing at local university	Coalition coordinator for a county department of health	Community needs assessment in rural areas
2	Assistant professor in nephrology at local medical school	Executive director for community-based organization focusing on diabetes prevention	Non-medical drivers of kidney disease in racial minority populations
3	Assistant professor in human development and family studies at local university	Program executive of health and wellness at YMCA	Physical fitness barriers among older adults post-pandemic
4	Assistant professor in ethnic studies at local university	Executive director for LGBTQ+ advocacy organization	Housing experiences of LGBTQ+ populations
5	Assistant professor in pediatrics at local medical school	Executive director for community-based organization focused on supporting Black mothers	Maternal health in African American populations

### Organization of CAP-HEP program

Selected community-academic partnership teams participated in the CAP-HEP Program during a 12-month period starting in the summer of 2023, with program activities organized as follows: (a) an introductory 2.5-day short course, (b) bimonthly trainings based on areas of interest identified during the 2.5-day short course, (c) bimonthly one-on-one sessions with CAP-HEP mentors, and (d) a closing 1-day session. The first goal of these activities was to provide foundational, structured training to academic and community partners to engage in health equity research. An emphasis was placed on CBPR and collaborative partnerships. A second goal was to maximize the value, scalability, and dissemination of equity research by giving the community a stake in the process. Third, we aimed to promote and produce rigorous, community centered research on health equity, and to do so through ongoing mentoring and instruction. A fourth goal was to facilitate cross-campus and regional collaborations, through formal group instruction and networking opportunities. Finally, we aimed to build capacity and diversify the workforce of equity researchers working alongside community entities.

#### The short course

We developed an 11-page participant manual to align short course activities with goals and resources. A 12-page facilitator manual also contained activity instructions, facilitation instructions, and required materials. An agenda was distributed every day and relevant literature was distributed at the end of the program through a shared folder. At the end of each day and of the 2.5-day short-course the CAP-HEP team administered an anonymous online survey assessing participants’ satisfaction with the program’s activities and increased knowledge and skills related to learning objectives.

The sessions in the short course consisted of a welcome dinner at a restaurant, experiential learning activities (16), team strategic planning, didactic lectures, and invited panel presentations (Table 2). Experiential learning activities, such as The River of Life (12), a power “construction and deconstruction” activity for faculty using legos, and others, focused on providing a safe environment where participants could explore the values committing them to their partnership, take perspective on their shared and individual journeys, and to encourage agency and promote appreciation for each other (16). Participants also had planning sessions where they could apply key CBPR concepts to their individual projects. Supplementing experiential learning activities and team planning sessions were skill-building didactic lectures on topics, such as photovoice, world cafe conversations, and coalition-building, among others (17–19). Finally, panel discussions featured seasoned community-academic teams and were co-facilitated by academic and community leaders with a strong history of collaboration in the region. Such co-facilitated sessions were intended to demonstrate successful collaborations and their paths, challenges, and opportunities.

**Table 2:** Organization of the CAP-HEP short course.

Day	Topics	Format
Day 1 Morning **Getting to know each other**	Introduction to short course	Academic facilitator discussion
	Team introductions	All teams circle
	Partnership journey: river of life activity	Team reflection and art
Day 1 Afternoon **Introduction to CBPR**	Working with diverse communities	Academic facilitator presentation
	CBPR principles and CBPR in action	A-C Co-facilitator presentation
	Team reflection	Team reflection
Day 2 Morning **Developing partnerships**	Partnership trust-building, formation, and sustainability	Moderated A-C panel
	CBPR toolkit	Academic facilitator presentation
	Project planning-teams	Team reflection and strategic planning
	Model partnerships	Keynote A-C speakers
Day 2 Afternoon **Implementing projects**	A-C tracks focused on partnership power dynamics	Researcher discussion; Community member discussion
	Project planning-teams	Team reflection and strategic planning
Day 3 Morning **Evaluating projects and evaluating partnership**	Disseminating and scaling projects	Moderated community panel discussion
	Project evaluation	A-C presentation
	Funding strategies	Funder presentation
	Next steps and closing	Academic facilitator discussion

A-C, academic-community.

#### Seed funding

Starting in the fall of 2023—3 months after the short course began—we met monthly with participating teams to support the development of each team’s proposal for seed funding. The proposal included a 2-page application and logic model, to be approved for seed funding. Teams worked with their mentors to develop their idea and prepare the proposal. All partnerships received $2,500 in seed funding, with four of the teams successfully completing their project by the end of the 12-month period.

#### Monthly peer mentoring and learning sessions

CAP-HEP teams met monthly starting in the fall of 2023, alternating between bi-monthly sessions with all mentors and partnership teams and bi-monthly didactic sessions. Mentor and partnership team meetings focused on reinforcing the principles and tools taught during the short course, exposing participants to new tools and advanced topics. Half of the sessions were hosted in person, while the other half were hosted virtually for convenience. In didactic sessions, the CAP-HEP leadership team presented webinars on a variety of CBPR concepts and strategies. Topics were identified by participating community-academic teams throughout the short course and often included a deep dive into a topic that had been covered during the short course and that they wanted to implement in their project. This included the following topics: community advisory boards; world café conversations; community engagement studios; survey design; qualitative data collection and analysis; and photovoice. In addition, participating teams provided project updates and engaged in real-time peer mentorship and troubleshooting on a bimonthly basis. These peer mentoring sessions fostered networking and community-building among and between teams.

Several of the monthly learning sessions utilized experiential learning similar to the short course. For example, one in-person session was focused on planning and implementing a world café conversation (WCC), where CAP-HEP participating teams co-moderated sessions alongside CAP-HEP mentors. This WCC session focused on community leaders’ expectations of research and community engagement from the local university and medical school. Primary questions included: (a) What is impactful research from the perspective of patients and other community stakeholders? (b) What health topics should researchers at [our academic institution] be focused on? (c) How should community be involved in research? and (d) What barriers or challenges prevent community partners from participating in research? Nineteen local community leaders we had previously worked with, and who were external to the program participants, volunteered to participate in the WCC, providing an opportunity for CAP-HEP partnership teams to learn how to use innovative community engagement methods beyond traditional focus groups. Through the WCC, teams practiced presenting a research idea, gathering community input through a dynamic Q&A session, and reporting back findings—all within a single session. Notably, one of the CAP-HEP teams utilized WCCs to assess fitness needs and preferences of low-income older populations currently enrolled at the YMCA, and a comparable group not enrolled.

Additionally, one of the virtual peer mentoring sessions focused on photovoice to engage and mobilize community action and included a brief lecture as well as dedicated time to practice the method themselves. Subsequently, a CAP-HEP team utilized photovoice to capture Black mothers’ parenting experiences. A third team created a video with oral histories about geographical displacement and its effects on healthcare access, which they intended to post on the non-profit website of the community partner and present to local lawmakers. Most teams utilized qualitative methods, which was another topic covered in the bi-monthly sessions.

#### Mentoring one-on-one sessions

Every other month starting in the fall of 2023, CAP-HEP teams met with a pair of CAP-HEP mentors, one from the local university and another a community leader. Academic and community co-mentors were selected based on past or active collaboration that they could draw upon during the mentoring process. These meetings were virtual and were designed to create a space for individual partnership teams to plan their projects at length and gather input from experienced academic-community partners. Topics of discussion typically included proposal development, project implementation, quantitative or qualitative analysis support, and evaluation. Capacity-building and partner collaboration and equitable use of resources were also central to these meetings.

#### Closing session

In the summer of 2024, at the end of the 12-month mentoring program, the five partnership teams met in person with the CAP-HEP leadership team and their academic and community mentors to present the process and outcomes of their projects, to reflect on their experiences with the program, and to receive a certificate of completion. During the closing session, CAP-HEP teams completed a brief online survey, and they engaged in a reflection exercise that identified program areas of strength and suggestions for improvement, as well as what they learned about CBPR and themselves throughout the year-long program. They also identified accomplishments and lessons learned. This meeting culminated in a group photo and a celebratory reception at a local restaurant. These evaluative components were approved by our institutional review board.

## Results

### Team composition

Five community-academic partnership teams participated and completed the CAP-HEP program (Table 1). These teams represented ***four schools,*** three from the same institution of higher education (i.e., Medical School, College of Liberal Arts, College of Natural Sciences) and one from a different institution of higher education (i.e., Nursing) and ***five departments*** across both institutions (i.e., Internal Medicine, Pediatrics, African and African Diaspora Studies, Human Development and Family Sciences, and Nursing). All academic partners were at the Assistant Professor level. The community organizations served ***five low-income populations** with intersecting identities* (i.e., individuals with chronic health conditions, Black mothers, BIPOC LGBTQ individuals, older adults and rural residents) and the team projects reflected five ***project focus areas*** (i.e., gentrification and healthcare access, gender-affirming care, perinatal mental health, rural health, and physical activity for older adults).

### Project activities and accomplishments

All partnership teams reported their progress at a final presentation. Team projects were the foundation for other initiatives. For example, one team created an oral history documentary that has since guided the community partner’s non-profit organization’s advocacy on displacement in Central Texas. Another team that focused on small towns and rural communities outside a metropolitan area conducted focus groups and interviews with community leaders from those communities. They subsequently used the findings to complement their county’s community health improvement plans, which until then had not assessed needs in those communities. The partnership team went on to publish this work in a peer-reviewed journal, and the community partner successfully applied for a doctoral program in community development. CAP-HEP mentors were invited to serve as co-authors on the article and to prepare letters of recommendation for the community member’s doctoral application.

### Short course evaluation

Overall, participants provided positive feedback on the 2.5-day short course. Most participants reported that the course was excellent or very good (83%; *n* = 5) and strongly agreed (67%; *n* = 4) that they learned CBPR tools that they could apply to their work. Participants reported enjoying the experiential activities that allowed them to review their work and progress thus far, identify opportunities for growth, as well as generate possible project ideas. In an open-ended question about overall impressions, one participant wrote, “Thank you so much for offering this opportunity; I feel less alone in embarking on new CBPR-related projects.”

We also received constructive feedback on how to improve future training in the short course, such as providing more time for discussion; providing pre-work or readings about key CBPR concepts or best practices for community engagement; and discussing more the historical context regarding harm and distrust in the community due to unethical healthcare or research practices. Based on this feedback, we created a shared folder with a list of readings and resources, organized by topics such as roles for researchers, defining community, conceptual frameworks, case studies, maintaining and assessing partnership and dissemination of findings. We also incorporated group discussion in monthly learning sessions, so teams could benefit from peer mentoring as well as didactic lectures.

### Yearlong evaluation

#### Program strengths

In a post-program evaluation conducted at the conclusion of the 12-month capacity-building program, participants reported that the CAP-HEP program was excellent (70%; *n* = 7), very good (20%; *n* = 2) or good (10%; *n* = 1). Most participants (90%; *n* = 9) reported that the CAP-HEP program was extremely useful or very useful and 100% (*n* = 10) of participants strongly agreed or agreed that mentor meetings were helpful.

When asked about the didactic bimonthly learning sessions, 100% of participants reported that all sessions were somewhat or very relevant to their work. Sessions that were broadly focused on research skills such as qualitative data collection and analysis and survey design were considered very relevant, while sessions that were focused on a particular methodology like photovoice or community engagement studios were considered somewhat relevant. Participants reported several accomplishments including the submission (60%; *n* = 3 teams) or receipt (40%; *n* = 2 teams) of grant funding. Several teams (60%; *n* = 3 teams) also reported presenting at local, state or national conferences. One team submitted a manuscript, which was published in a peer-reviewed health journal. An advantage of participants being local is that they sought collaboration with CAP-HEP mentors beyond their partnership projects, noted in 75% (*n* = 4 teams) of the teams, with two active ongoing collaborations underway and a third collaboration completed.

Participants also highlighted the importance of forming close relationships with other teams and having a space to discuss challenges associated with conducting CBPR during monthly learning sessions. Individuals reported facing outside pressures, such as balancing research with other academic expectations (academic) or competing priorities (community), that they were able to discuss during peer mentoring sessions. Participants also described learning more about community organizations and resources, and planning to leverage these new relationships for future partnerships.

#### Program areas of improvement

Participants provided several recommendations for future iterations of the 12-month CAP-HEP. First, they suggested additional didactic sessions on IRB procedures, funding and sustainability, and grant writing. Academic and community members from participating teams noted that while obtaining IRB approval was necessary, the process was time-consuming and required familiarity navigating a complex submission platform—an especially burdensome task given the scale of and funding for the project. Second, although a community strategy team had been consulted in the planning of the program, they recommended including community participants more in the planning of the program, and suggested they become part of an ongoing advisory group to support future cohorts. Anecdotally, we observed that while CAP-HEP strengthened relationships between community members and academic institutions, more work needs to be done to address longstanding negative perceptions of research, in general, and research institutions, based on historical abuses. One community partner voiced skepticism that academic-community partnerships could be equitable and provided suggestions for the program to further elevate community members’ role within the partnership. Suggestions included compensating community members for their time spent on the program and being mindful of the time it took for them to coordinate and implement their project, in light of their regular employment and ongoing community service obligations.

## Discussion

The 12-month CAP-HEP capacity-building program addresses the growing need for academic and academic medical institutions to build robust infrastructure for CBPR—particularly efforts that position community organizations as equal partners. By supporting teams composed of both academic and community members, CAP-HEP not only promises to incentivize researchers to pursue CBPR and develop the skills essential for success, but also to introduce community partners to academic methodologies that can strengthen the implementation and evaluation of their programs, ultimately enhancing reach and impact.

Participants from our first cohort of five teams found the various CAP-HEP program components to be valuable. Specifically, they reported that (a) the experiential learning activities in the short-course solidified their partnership commitments, (b) the skills-focused content in the peer-mentoring sessions developed their shared research project, and (c) the seed funding and mentored experiences facilitated ongoing success. Notably, the benefits of their participation went beyond individual teams. Although we limited participation to local teams for cost reasons, an unexpected advantage of this approach was that teams were able to build relationships across research areas and community organizations. This fostered a local community of like-minded individuals, broadened their networks, and for many, led to cross-team collaboration and sustained engagement with mentors. Teams also developed understanding of how to bridge the worlds of academia and community, which have historically felt separate. One community member felt so drawn to the benefits of bridging these worlds that they have successfully entered a doctoral program since completing CAP-HEP. Others used their projects to develop community-based initiatives, secure funding, publish and disseminate their work.

Experiential learning has been the hallmark of other community engagement capacity-building programs (2, 12, 16), as it promotes capacity for perspective-taking and vulnerability when working with people of different backgrounds (15). CAP-HEP drew on experiential learning to assist community-academic teams in identifying each partner’s assets and growth areas and reflect and plan for partnership processes more broadly. Experiential activities were mostly taught as part of the short course, and included values exploration and partnership history, partnership planning and expected outcomes, discussion of power dynamics in democratizing research, and separate discussions for academic and community partners about challenges they face in their work environment. For example, faculty engaged in an experiential activity involving legos, where they were instructed to physically construct and represent their place in academia and the communities in which they wish to engage. This is significant because researchers interested in CBPR often face contradictory messages and demands in their academic environments. Our exercise aimed to provide a safe place where they could acknowledge the lack of agency they at times experience in academia due to their area of scholarship, approach to scholarship, or their intersecting identities, while also recognizing the power they have as academics relative to their community partners (20).

Whereas our program mirrored others in its reliance on experiential learning (16), especially as part of the short course, we incorporated a heavy emphasis on skills-building in community engagement and research. We recognized that to carry out a community-based participatory research project successfully, early career faculty in the program needed to build competence in CBPR and mixed-methods, while community leaders could benefit from structured and innovative approaches to engagement and evaluation. Skills-building content requested by teams at the conclusion of the short course, was delivered by our CAP-HEP faculty and staff trainers, who have expertise in community engagement tools such as photovoice, world café conversations, community engagement studios, and others, but who also have extensive experience conducting quantitative and qualitative research as part of collaborative projects with local and regional partners. Participating teams learned through lectures, hands-on application, and by incorporating these methods into their team projects. For example, one team utilized world café conversations to engage older populations, another team conducted photovoice with Black mothers, yet a third team utilized surveys, and the other two conducted focus groups and interviews.

Whereas faculty may have access to research training in their academic setting, our approach to research training is that data collection, whether quantitative or qualitative, is not conducted in a vacuum. That is, data collection that involves engagement and trust-building through community partnership is in the interest of ethical and effective research (21). Moreover, data collection shifts away from an objective examination of a subject that further “makes the research distant from the people” (p. 80), to co-creation of knowledge that humanizes their life experiences, dignifies the person, and builds hope and agency (15). Noteworthy is that participating faculty were in their early career stages, so they had limited training and exposure to these methods in a community context. Similarly, the clinical faculty members had limited research experience, so the benefit of these specific skill-building tools extended to both community and academic team members.

As we plan for future cohorts, we heed this first cohort’s recommendations for improvement. First, we plan to increase skills-based research training offered through CAP-HEP, including basic quantitative and qualitative skills, IRB submissions, as well as more nuanced training in partnership sustainability. Second, although we incorporated equitable practices throughout the CAP-HEP program, such as co-facilitating sessions with community collaborators, having community collaborators as co-mentors, and discussing issues of power in experiential learning activities, ongoing conversations with the teams suggests that more needs to be done to build trust. We attribute this to the historical abuses committed by academic institutions that range from “parachute research” and neglect, extractive language and power dynamics with academics as “knowledge producers” and communities as “knowledge recipients,” to downright deceit and harmful practices (15). While community participants felt empowered by the program and in their partnership, they also described challenges related to time, training location, and financial constraints—factors that reinforced existing inequities. Looking ahead, we plan to address these concerns through both content and process-level changes. For example, we aim to develop new program content that includes deeper conversations about community burden in CBPR, trust-building, and equitable resource sharing. Process-wise, we plan to implement more intentional practices such as forming a dedicated community-academic decision-making board or steering committee and proactively addressing the financial and time burdens placed on community members. These actions may include offering stipends for participation, rotating session facilitation across community sites, and scheduling program activities with greater sensitivity to participants’ availability.

Community involvement in the planning and implementation of CAP-HEP could be strengthened. During the early phases, we sought input from our institution’s Community Strategy Team to guide planning and community outreach. We also held quarterly meetings with CBPR Academy consultants from the Detroit URC, whose community partners were instrumental in the original design of the CBPR Academy upon which our model was based, and whose consultants’ community-rooted expertise helped shape the broader context of our work. While their community insights were valuable during discussions, the consultants who engaged over the two-year period were academic researchers and administrative staff, largely due to time constraints faced by their community partners. We believe that community perspectives were meaningfully integrated from multiple sources; however, we recognize that more direct and sustained involvement from community members would have further strengthened the training model and more fully aligned with CBPR principles of co-creation. For future cohorts, we will create a decision-making advisory board or steering committee, to include many of the participants from our first cohort, to help us identify more opportunities for incorporating equitable practices into CAP-HEP.

## Conclusion

We provided dedicated training in CBPR practices for community practitioners and academics. The CAP-HEP program built capacity for academic-community partners who were engaged in health equity research and initiatives. Using a combination of didactic and experiential learning opportunities, in addition to peer and formal mentorship, dyads were able to collaborate more effectively, and participants gained essential skills. Importantly, faculty developed a better understanding of how to address barriers to community health research, and community members developed an interest in research and evaluation, as well as an increased appreciation for academic institutions. Ideally, future-funded studies over multiple cohorts will be able to more rigorously measure improvement in CBPR research knowledge, accomplishments and impact, and partnership development and quality.

## Data Availability

The raw data supporting the conclusions of this article will be made available by the authors, without undue reservation.
